# Ouabain-mediated downregulation of ALKBH5 and IGF2BP2 inhibits the malignant progression of DLBCL

**DOI:** 10.3389/fphar.2024.1447830

**Published:** 2024-08-30

**Authors:** Yuxin Hong, Hehua Ma, Haoyi Yang, Yuning Zhu, Yuan Wei, Zhenzhen Xu, Yuwen Zhang, Dandan Jin, Zhiyou Chen, Wei Song, Juan Li

**Affiliations:** ^1^ Department of Phase I Clinical Trials Unit, Nanjing Drum Tower Hospital Clinical College of Nanjing University of Chinese Medicine, Nanjing, China; ^2^ Phase I Clinical Trials Unit, Nanjing Drum Tower Hospital, Affiliated Hospital of Medical School, Nanjing University, Nanjing, China; ^3^ Department of Phase I Clinical Trials Unit, China Pharmaceutical University Nanjing Drum Tower Hospital, Nanjing, China

**Keywords:** m^6^A, ALKBH5, IGF2BP2, ouabain, DLBCL

## Abstract

m^6^A modification is a crucial epigenetic regulatory mechanism in diffuse large B-cell lymphoma (DLBCL). Low-dose cardiotonic drugs have been shown to induce apoptosis in DLBCL cells through epigenetic modulation. However, the involvement of the cardiotonic drug ouabain in the malignant progression of DLBCL remains unclear. Our study revealed that ouabain indeed contributes to the malignant progression of DLBCL through m^6^A modification. Through qPCR analysis, we observed a negative correlation between ouabain concentration and the expression levels of the demethylase ALKBH5 and the m^6^A-binding protein IGF2BP2 in DLBCL cells. Furthermore, high expression levels of ALKBH5 and IGF2BP2 were identified in both the GEO database and DLBCL patient tissue samples. Notably, elevated ALKBH5 and IGF2BP2 promoted cell proliferation both *in vitro* and *in vivo*. Inhibition of their expression rendered DLBCL cells more sensitive to ouabain treatment, resulting in significant suppression of cell proliferation, G1/S phase cell cycle arrest, and increased apoptosis. In summary, our results clarify that the demethylase ALKBH5 and the m^6^A-binding protein IGF2BP2 are involved in the malignant progression of DLBCL, and that the cardiotonic drug ouabain can inhibit the proliferation of DLBCL cells by inhibiting the expression of ALKBH5 and IGF2BP2, which provides new insights into the targeted treatment of DLBCL.

## 1 Introduction

Diffuse large B-cell lymphoma (DLBCL) is an aggressive malignancy and ranks as one of the most common non-Hodgkin’s lymphomas worldwide (Takahara et al., 2023). The current standard treatment for DLBCL consists primarily of R-CHOP therapy, which includes rituximab, cyclophosphamide, doxorubicin, vincristine, and prednisone (Sehn and Gascoyne, 2015). Approximately 60% of patients achieve complete remission with R-CHOP therapy (Sehn et al., 2021), but owing to the high heterogeneity of DLBCL, approximately 40% of patients experience posttreatment relapses, leading to unfavorable treatment outcomes (Sarkozy and Sehn, 2018). Therefore, there is an urgent need to develop a new combination therapy to address this clinical challenge. It is now widely accepted among clinicians that adding new drugs (preferably less toxic) to the standard R-CHOP regimen is necessary for treating relapsed and refractory DLBCL patients (Chiappella et al., 2017). Consequently, the discovery of targeted drugs for first-line DLBCL treatment and the identification of new molecular targets are crucial for improving treatment efficacy.

As a representative cardiotonic drug, ouabain is commonly used to treat heart failure and atrial fibrillation. Although there have been attempts to treat tumors with cardiac glycosides (Shen et al., 2019), their use has been limited because of the difficulty in determining the effective therapeutic dose and the lack of understanding of their mechanism of action (Prassas and Diamandis, 2008).In the 1970s, Stenkvist reported that patients with breast cancer who took cardiac glycosides (digoxin) for heart disease presented more benign changes in tumor tissues than those who did not take digoxin. The recurrence rate and mortality rate of patients with breast cancer after surgery are also significantly lower among those taking digoxin (Stenkvist et al., 1980). These findings led researchers to investigate the potential tumor-suppressing effects of cardiac glycosides. In 2001, Haux et al. investigated whether patients taking digitoxin for cardiac disease have a different cancer incidence than does the general population. They reported that as the blood concentration of digitalis increased, the incidence of leukemia, lymphoma, and urinary system tumors decreased (Haux et al., 2001). Previous studies have shown that ouabain can inhibit proliferation in acute myeloid leukemia (AML), and promote tumor cell apoptosis in acute T lymphoblastic leukemia (Valdes et al., 2007; Tailler et al., 2011). These findings suggest that ouabain may play a role in various hematologic tumors including DLBCL, suggesting further exploration into its possible function and mechanism in DLBCL is needed.

In the past decade, many epigenetic modifiers have been developed and applied in the clinical treatment of patients with hematological tumors (Yankova et al., 2021; Weng et al., 2022; Chen et al., 2023a), but little has been explored for the treatment of DLBCL with epigenetic modifications (Sermer et al., 2019; Uddin et al., 2021). Through RNA sequencing and detection of m^6^A-modifying enzymes, we demonstrated that the cardiac glycoside drug ouabain affects DLBCL by influencing m^6^A methylation. m^6^A RNA methylation is the most common internal modification of mammalian mRNAs and plays an important biological role by regulating important cellular processes (Sun et al., 2019; Uddin et al., 2021). m^6^A modifications are dynamically regulated by methyltransferases, demethylases, and binding proteins, thereby determining mRNA fate by regulating mRNA stability, transport, translation, and degradation progression (Li H.-B. et al., 2017). qPCR further demonstrated that ouabain downregulated the m^6^A demethylase ALKBH5 and the m^6^A-binding protein IGF2BP2 in ouabain-treated DLBCL cells. ALKBH5 is a demethylase that affects nuclear RNA output, metabolism, and gene expression (Zhu et al., 2019). Human insulin-like growth Factor 2(IGF2) mRNA binding protein 2(IGF2BP2/IMP2) is an RNA-binding protein that regulates various biological processes (Bell et al., 2012). They are involved in the development of cancer by communicating with different RNAs such as microRNAs (miRNAs) (Li X. et al., 2017), messenger RNAs (mRNAs) (Dai et al., 2017), and long noncoding RNAs (lncRNAs) (Wu et al., 2018; Wang et al., 2021). Increasing evidence shows that ALKBH5 and IGF2BP2 are closely related to tumor growth, proliferation, and survival. Inhibition of ALKBH5-mediated m^6^A modification decreases USP1 expression, which can ameliorate glucocorticoid resistance in T-ALL (Ye et al., 2023). As m^6^A-binding proteins, IGF2BP2 and IGF2BP3 increase the stability of DDX21 in a m^6^A-dependent manner, leading to the progression of AML (Liu et al., 2022). However, the role of ALKBH5 and IGF2BP2 in DLBCL tumorigenesis and their molecular mechanisms remain unclear.

It has been reported that ouabain regulates tumor suppressor genes via epigenetic silencing (Raynal et al., 2016). Ouabain can also suppress the ability of a cancerous lesion to spontaneously shed CTC clusters by inhibiting Na^+^/K^+^ ATPase *in vivo*, leading to a remarkable reduction in metastasis seeding ability (Gkountela et al., 2019). These findings undoubtedly reveal the possibility of an antitumor effect of ouabain through the regulation of epigenetic factors. Therefore, this study aimed to investigate the downregulation of the m^6^A demethylase ALKBH5 and m^6^A-binding protein IGF2BP2 induced by treatment with the cardiac glycoside drug ouabain, which inhibits the proliferation and promotes apoptosis of DLBCL cells. Based on these findings, the key effective molecules of ouabain in the treatment of DLBCL through epigenetic modification were identified, which provides a theoretical and experimental basis for clinical guidance for the combination of DLBCL with other drugs to improve poor patient prognosis.

## 2 Materials and methods

### 2.1 Cell lines and reagents

The human cell lines SU-DHL4, OCI-Ly3, SU-DHL2, and U2932 were cultured in RPMI-1640 medium supplemented with 10% fetal bovine serum, 100 U/mL penicillin, and 100 μg/mL streptomycin (Invitrogen, Carlsbad, CA, United States) under an atmosphere with 5% CO_2_ at 37°C. Ouabain was purchased from Sigma-Aldrich (St. Louis, MO, United States).

### 2.2 Tissue samples

A total of 7 tissue samples from DLBCL patients and 3 tissue samples from patients with lymph node hyperplasia were obtained from Nanjing Drum Tower Hospital. All the tissues were immediately stored at −80°C. Informed consent was obtained from each patient, and all samples were collected with informed consent and approved by the Medical Ethics Committee of the Affiliated Hospital of Nanjing University Medical School.

### 2.3 RNA interference and lentiviral infection

The ALKBH5, shALKBH5, or empty vector lentiviral plasmids were purchased from GeneChem (Shanghai, China) and the IGF2BP2, shIGF2BP2, empty vector lentiviral plasmidsandsmall interfering RNAs (siRNAs) were purchased from GenePharma (Suzhou, China). 48 h after transfection, cell lines were treated with 2 μg/mL puromycin for 2 weeks and screened for cell lines stably overexpressing or silencing ALKBH5/IGF2BP2. The primer sequences are shown in Supplementary Table S1.

### 2.4 Quantitative PCR

Total RNA from cells or frozen tissues was isolated via TRIzol reagent (Invitrogen, Carlsbad, CA, United States) and used as a template to synthesize cDNA via a reverse transcription kit (Takara, Dalian, China). Quantitative PCR (qPCR) was performed via SYBR Green reagents (Vazyme, Nanjing, China), with β-actin used as an internal control. The primer sequences are shown in Supplementary Table S2.

### 2.5 Western blot analysis

The protein concentration of the cell lysates was quantified via a bicinchoninic acid protein assay kit (Beyotime, Jiangsu, China). Equal amounts of protein were analyzed by immunoblotting with an anti-ALKBH5 antibody (#D264377, BBI), an anti-IGF2BP2 antibody (#D120813, BBI), an anti-SF3B4 antibody (Catalog #10482-1-AP, Proteintech), and an anti-YWHAG antibody (12381-1-AP, Proteintech),with an anti-β-actin antibody (Sigma-Aldrich, United States) used as an internal control.

### 2.6 Cell viability and proliferation assays

To calculate the IC50 values of ouabain, SU-DHL4, OCI-Ly3, SU-DHL2, and U2932 cells were seeded into 96-well plates at a density of 3 × 10^4^ per well, treated with different concentrations of ouabain (0, 30, 60, 90, 120, 150 or 180 nM) and cultured for 24 h. Cell viability was measured by adding Cell Counting Kit-8 (CCK-8; Vazyme, Nanjing, China) to each well. The absorbance was measured at 450 nM by spectrophotometry after incubation for 3 h at 37°C.

Cell proliferation was evaluated via a Cell Counting Kit-8 (CCK-8; Vazyme, Nanjing, China). The cells (3 × 10^4^ per well) were treated with ouabain (SU-DHL4 at 80 nM; OCI-LY3 at 140 nM; U2932 at 140 nM) in triplicate wells of a 96-well plate and incubated at 37°C. CCK-8 reagent was added to the wells at 0 h, 24 h, 48 h, and 72 h. Subsequently, the absorbance values of the samples were measured at 450 nm.

### 2.7 Cell cycle analysis

After 48 h of transfection, the cells were first harvested, washed twice with PBS and incubated in PBS containing 0.02% Triton X-100, 0.1 mg/mL RNase (Sigma-Aldrich), and 10 μg/mL propidium iodide (PI, 40%, Sigma-Aldrich) for 30 min at 37°C. The cell cycle distribution was detected via a FACScan flow cytometer (Becton Dickinson & Co., San Jose, CA, United States).

### 2.8 Apoptosis assay

The cells (5 × 10^5^ cells per well) were seeded in 24-well plates and incubated with ouabain at the IC50 for 24 h. Apoptosis was examined via flow cytometry on a FACScan flow cytometer (Becton Dickinson & Co., San Jose, CA, United States) after the cells were stained with Annexin V-PE and 7-AAD.

### 2.9 Bioinformatic analysis

The gene expression profiles were obtained from Gene Expression Profiling Interactive Analysis (GEPIA) and the Gene Expression Omnibus (GEO, GSE12453; GEO, GSE83632).

### 2.10 RNA-sequencing analysis

RNA sequencing analysis of the differentially expressed enriched genes of OCI-LY3 cells treated with ouabain was performed via GO and KEGG analyses. RNA sequencing was performed by Lc-Bio Technologies (Hangzhou, China).

### 2.11 Statistical analysis

Student’s t-test (two-tailed) was performed to evaluate differences between two groups. One-way analysis of variance (ANOVA) was used to evaluate differences between more than two groups. A minimum of three replicates were conducted in all the experimental trials, and the results are reported as the means ± standard deviations (SDs). Differences were considered significant when the *P*-value was <0.05 (*p < 0.05, **p < 0.01, ***p < 0.001, ****p < 0.0001). Statistical analyses were performed via GraphPad Prism 8.0.

## 3 Results

### 3.1 Establishment of the IC50 for each cell line treated with ouabain

**FIGURE 1:**
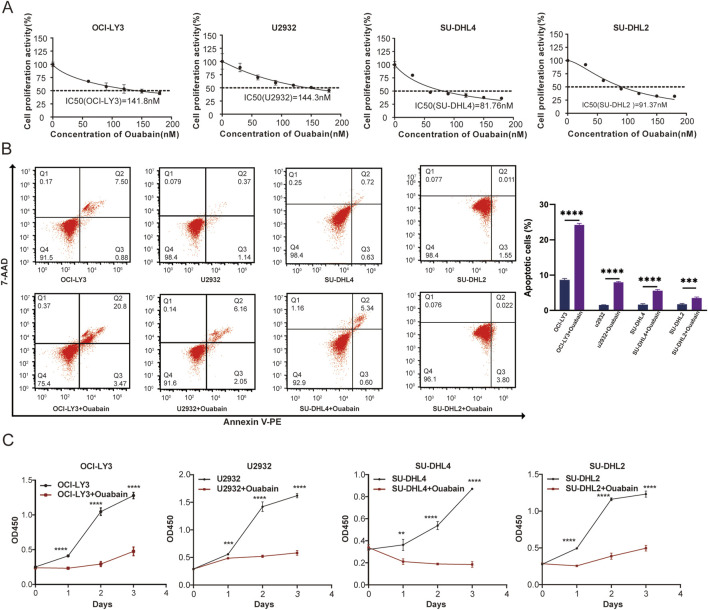
The viability of the four DLBCL cell lines was determined following treatment with different concentrations of ouabain. **(A)** The viability of four DLBCL cancer cell lines treated with different concentrations of ouabain was measured via a Cell Counting Kit 8 assay (CCK-8). IC50, 50% inhibitory concentration. **(B)** Representative flow cytometric images of apoptosis in four DLBCL cell lines after treatment with ouabain. Apoptosis was detected by Annexin V-PE and 7-AAD. **(C)** A Cell Counting Kit 8 assay was utilized to assess the proliferation of DLBCL cells (OC-LY3, U2932, SU-DHL4, and SU-DHL2) treated with ouabain. The data are presented as the means ± SDs; n = 3. *p < 0.05, **p < 0.01, ***p < 0.001, ****p < 0.0001 (Student’s t-test).

To investigate the impact of ouabain on DLBCL cell viability, we exposed DLBCL cells to varying concentrations of ouabain. The IC50 values of ouabain in OCI-LY3, U2932, SU-DHL4, and SU-DHL2 cells were 141.8 nM, 144.3 nM, 81.76 nM and 91.37 nM, respectively (Figure 1A). Furthermore, upon treating four DLBCL cell lines with ouabain at the IC50, we observed significant induction of cell apoptosis (Figure 1B). To further assess the ability of ouabain to effectively prevent the proliferation of DLBCL cells, we treated DLBCL cells with ouabain and observed their proliferation for three consecutive days (Figure 1C). Our findings revealed substantial suppression of DLBCL cell proliferation under ouabain treatment, confirming the efficacy of ouabain in inhibiting DLBCL cell proliferation and promoting apoptosis at low concentrations.

### 3.2 Involvement of ouabain in RNA methylation progression in DLBCL

**FIGURE 2:**
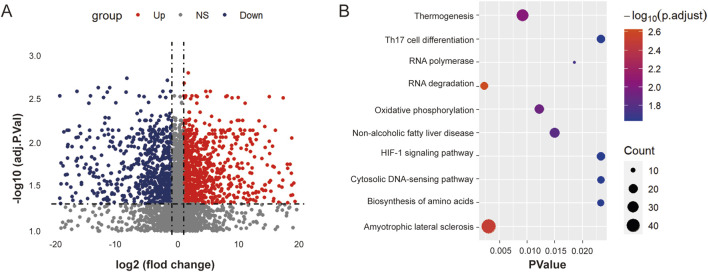
Ouabain is involved in the RNA degradation progression of DLBCL. **(A)** Heatmap displaying the differentially expressed genes between the control group and the ouabain treatment group. **(B)** KEGG Pathway analysis revealed that the RNA degradation signaling pathway was significantly enriched after ouabain treatment.

To clarify the role of ouabain in DLBCL, we first added ouabain to OCI-LY3 cells and conducted RNA-sequencing (RNA-seq) analysis to analyze the differentially expressed enriched genes (Figure 2A). KEGG pathway enrichment analysis revealed a close association between DLBCL cells and RNA degradation following ouabain treatment (Figure 2B). m^6^A methylation of RNAplays a crucial role in the degradation process. For example, YTHDF2 mediates mRNA degradation of tumor suppressors and induces AKT phosphorylation in an N^6^-methyladenosine-dependent way in prostate cancer (Li et al., 2020). As we all know, the miRNA-dependent mechanism also plays an important role in RNA degradation. The circular RNA circular RNA sponge for miR-7 (ciRS-7) inhibits the expression of UCHL1 to promote APP and BACE1 degradation by inhibiting the translation of NF-κB and inducing its cytoplasmic localization (Shi et al., 2017). Therefore, we aimed to explore whether ouabain-induced RNA degradation was caused by m^6^A modification or miRNA-dependent mechanism.

### 3.3 Effect of ouabain on RNA m^6^A methylation-related enzyme classes

**FIGURE 3:**
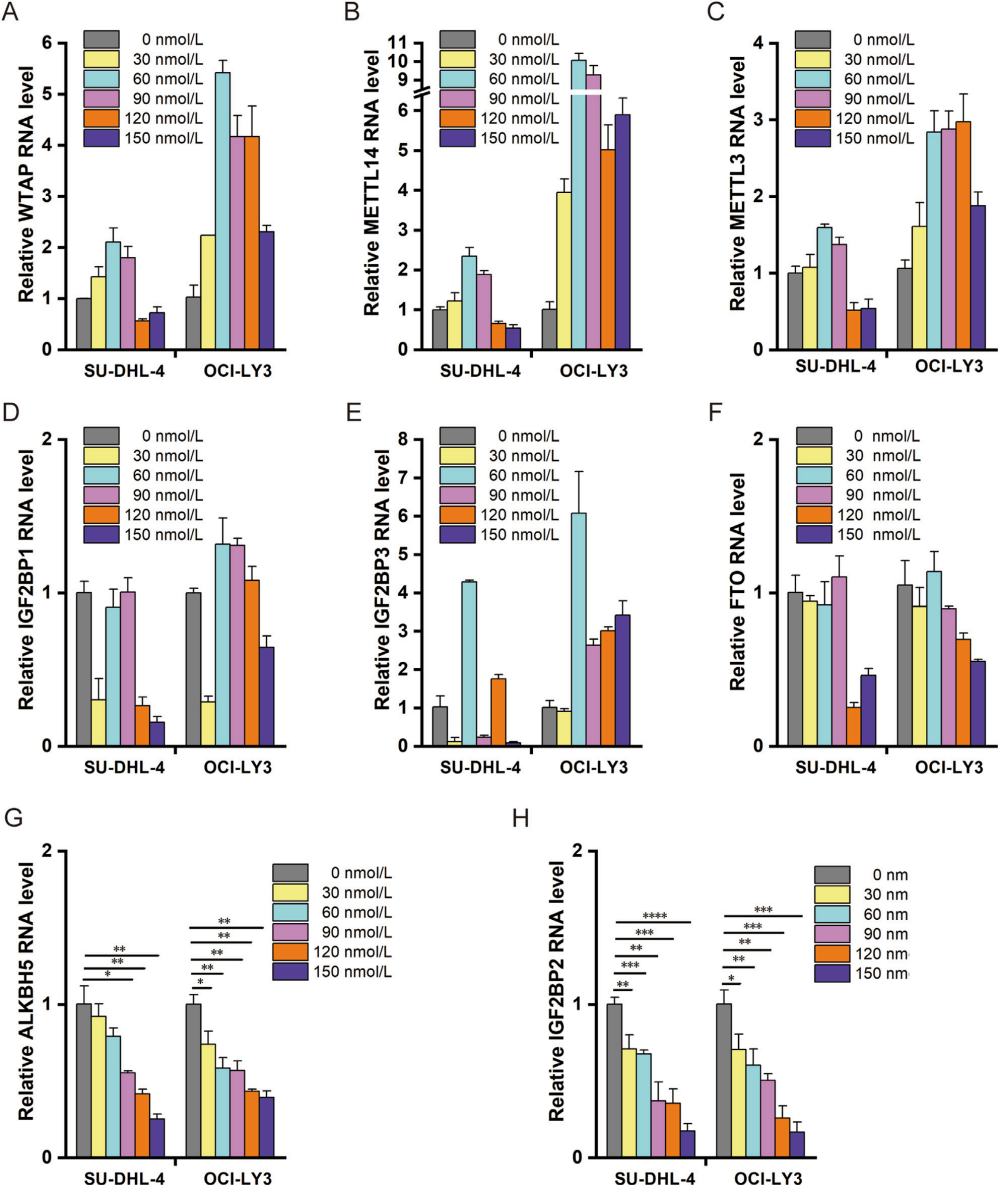
Effect of ouabain on RNA m^6^A methylation-related enzymes. **(A)** Effects of different concentrations of ouabain on the expression of the RNA m^6^A methylase WTAP were detected via q-PCR. **(B)** Effects of different concentrations of ouabain on the RNA m^6^A methylase METTL14 were detected via q-PCR. **(C)** Effects of different concentrations of ouabain on the RNA m^6^A methylase METTL3 were detected via q-PCR. **(D)** Effects of different concentrations of ouabain on the RNA m^6^A-binding protein IGF2BP1 were detected via q-PCR. **(E)** Effects of different concentrations of ouabain on the RNA m^6^A-binding protein IGF2BP3 were detected via q-PCR. **(F)** Effects of different concentrations of ouabain on the RNA m^6^A demethylase FTO were detected via q-PCR. **(G)** Effects of different concentrations of ouabain on the RNA m^6^A demethylase ALKBH5 were detected via q-PCR. **(H)** Effects of different concentrations of ouabain on the RNA m^6^A-binding protein IGF2BP2 were detected via q-PCR. The data are presented as the means ± SDs; n = 3. *p < 0.05, **p < 0.01, ***p < 0.001, ****p < 0.0001 (Student’s t-test).

To explore whether ouabain induces RNA degradation through m^6^A modification or through miRNA-dependent mechanisms, we detected changes in RNA m^6^A methyltransferases, demethylases, and reader enzymes in response to different concentrations of ouabain (Figure 3). The RNA demethylase ALKBH5 and the m^6^A-binding protein IGF2BP2 were most significantly regulated by ouabain, and their mRNA expression levels gradually decreased with increasing concentrations of ouabain, indicating an obvious gradient dependent relationship (Figures 3G, H). microRNA-induced silencing complex (miRISC) is a multi-protein assembly that uses microRNAs (miRNAs) to identify mRNAs targeted for degradation. The miRISCs contain the Argonaute (AGO) family protein AGO2, which plays an important role in the function of miRNA. To further explore whether AGO2 is involved in the mRNA degradation of ouabain-treated DLBCL cells, we performed mRNA half-life on LSM1 and DCP1A, which were enriched in the RNA degradation pathway after treatment with ouabain. We found that the half-life of LSM1 and DCP1A did not change significantly compared with the control after interference with AGO2 (Supplementary Figure S1A, B).On the other hand, it has been reported that miR-7-5p and miR-132-3p play RNA degradation roles in DLBCL(Morales-Martinez et al., 2020; Mansoor et al., 2023), so we further examined whether ouabain affects the RNA degradation process by regulating miRNAs in DLBCL. The experimental results showed that ouabain had no effect on the expression of miR-7-5p and miR-132-3p (Supplementary Figure S1C, D). These results showed that the miRNA-dependent mechanism did not play a role in ouabain-mediated RNA degradation. Therefore, we suggest that ouabain is involved in the progression of DLBCL by mediating m^6^A modification leading to RNA degradation, and ALKBH5/IGF2BP2 is a target molecule regulated by ouabain.

### 3.4 Gene expression analysis of ALKBH5 and IGF2BP2 in DLBCL tissues and cells

**FIGURE 4:**
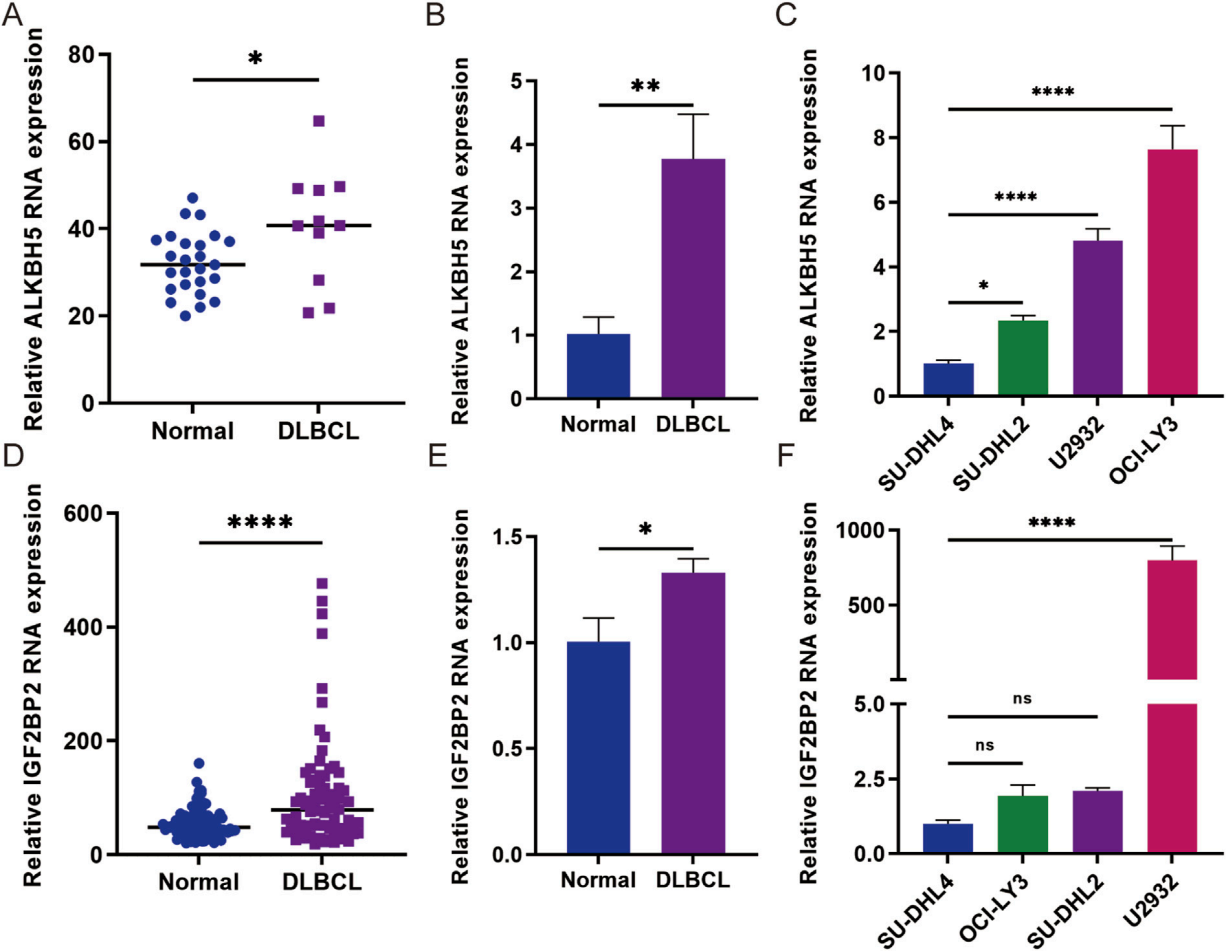
Gene expression analysis of ALKBH5 and IGF2BP2 in DLBCL tissues and cells. **(A)** The expression level of ALKBH5 in diffuse large B-cell lymphoma (DLBCL) was analyzed in the GSE12453 cohort. **(B)** Gene expression analysis of ALKBH5 in DLBCL tissues. **(C)** Gene expression analysis of ALKBH5 in DLBCL cells. **(D)** The expression level of IGF2BP2 in diffuse large B-cell lymphoma (DLBCL) samples from the GSE83632 cohort was analyzed. **(E)** Gene expression analysis of IGF2BP2 in DLBCL tissues. **(F)** Gene expression analysis of IGF2BP2 in DLBCL cells. The data are presented as the means ± SDs; n = 3. *p < 0.05, **p < 0.01, ***p < 0.001, ****p < 0.0001 (Student’s t-test).

To further investigate the expression of ALKBH5 and IGF2BP2, we searched the GEO database (GSE12453, GSE83632) and found that ALKBH5 and IGF2BP2 were highly expressed (Figures 4A, D). In addition, we collected 3 examples of normal lymph node tissues and 7 examples of diffuse large B-cell lymphoma tissues. qPCR confirmed that ALKBH5 and IGF2BP2 are highly expressed in DLBCL patients, and are associated with poor prognosis (Figures 4B, E). To select the appropriate cell model for each experiment, we detected the relative expression of ALKBH5 and IGF2BP2 in four DLBCL cell lines SU-DHL4, OCI-LY3, U2932, and SU-DHL2 (Figures 4C, F). Our findings revealed that ALKBH5 was relatively low in SU-DHL4 cells but relatively high in OCI-LY3 cells, whereas IGF2BP2 was relatively low in SU-DHL4 cells but relatively high in U2932 cells.

### 3.5 Knockdown of ALKBH5 reduced cell proliferation, and cell cycle arrest, and increased apoptosis in DLBCL

**FIGURE 5:**
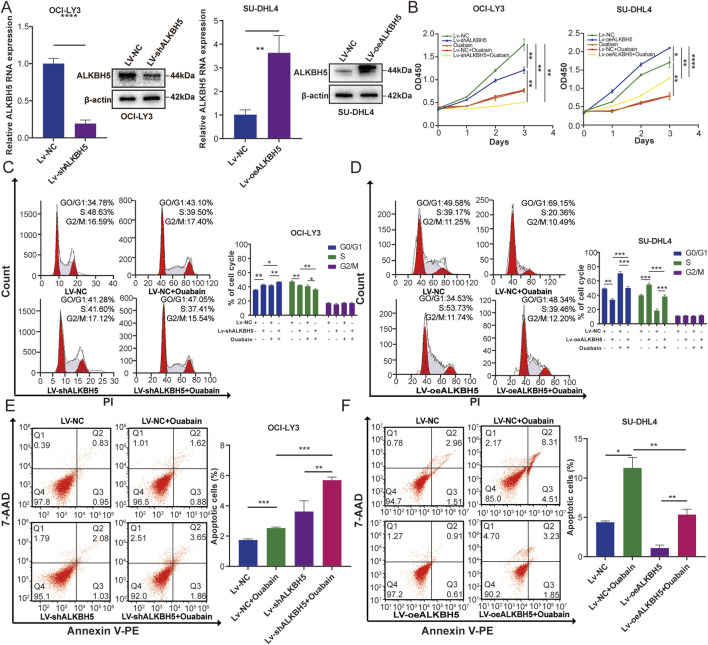
ALKBH5 affects the sensitivity of DLBCL cell lines to ouabain. **(A)** The knockdown effect of ALKBH5 in OCI-LY3 cells and the overexpression effect of ALKBH5 in SU-DHL4 cells were assessed via qPCR and protein expression of western blotting analysis (WB). **(B)** A Cell Counting Kit 8 assay was utilized to assess the proliferation of OCI-LY3 and SU-DHL4 cells treated with ouabain. **(C)** ALKBH5 knockdown combined with ouabain treatment significantly induced cell cycle arrest at the G1/S phase in OCI-LY3 cells, as assessed by flow cytometry. **(D)** ALKBH5 overexpression inhibited ouabain-induced cell cycle arrest at the G1/S phase in SU-DHL4 cells, as assessed by flow cytometry. **(E)** ALKBH5 knockdown combined with ouabain treatment significantly promoted apoptosis in OCI-LY3 cells, as assessed by flow cytometry. **(F)** ALKBH5 overexpression inhibited ouabain-induced apoptosis in SU-DHL4 cells, as assessed by flow cytometry. The data are presented as the means ± SDs; n = 3. *p < 0.05, **p < 0.01, ***p < 0.001, ****p < 0.0001 (Student’s t-test).

ALKBH5 has been reported to be upregulated in a variety of cancers including breast (Zhang et al., 2016), lung (Sun et al., 2022), and epithelial ovarian cancers (Nie et al., 2021), where it plays an oncogenic role in tumor progression. To assess the effect of ALKBH5 on DLBCL cells under the influence of ouabain, we overexpressed ALKBH5 in SU-DHL4 cells and downregulated ALKBH5 in OCI-LY3 cells (Figure 5A). These two cell lines were subsequently treated with ouabain. Proliferation experiments were conducted via a CCK-8 assay for the following groups: control group (Lv-NC), ouabain treatment group (Ouabain), ALKBH5 knockdown group (Lv-shALKBH5), combination of ouabain treatment and control group (Lv-NC + Ouabain), and combination of ouabain treatment and ALKBH5 knockdown group (Lv-shALKBH5+Ouabain) (Figure 5B left). As depicted in Figure 5B, ouabain decreased the viability of OCI-LY3 and SU-DHL4 cells when administered alone. Additionally, the overexpression of ALKBH5 significantly increased cell viability. Furthermore, the groups treated with combinations of ouabain and Lv-shALKBH5 exhibited a greater reduction in proliferation than did the group treated with ouabain alone. Flow cytometry analysis revealed that the cell cycle was significantly inhibited in the G1/S phase (Figure 5C). On the other hand, apoptosis was significantly greater in the Lv-shALKBH5+Ouabain group than in the other groups (Figure 5E). In contrast, when ouabain was applied to cells overexpressing ALKBH5, the CCK-8 method revealed that the inhibition of cell proliferation and cell cycle arrest was reduced in the Lv-oeALKBH5+Ouabain group, and cell apoptosis was reduced compared with that in the Lv-NC + Ouabain group (Figure 5B right, Figures 5D, F). These results suggest that downregulated ALKBH5 enhances the chemosensitivity of DLBCL cells to ouabain.

### 3.6 Knockdown of IGF2BP2 reduced cell proliferation, and cell cycle arrest, and increased apoptosis in DLBCL

**FIGURE 6:**
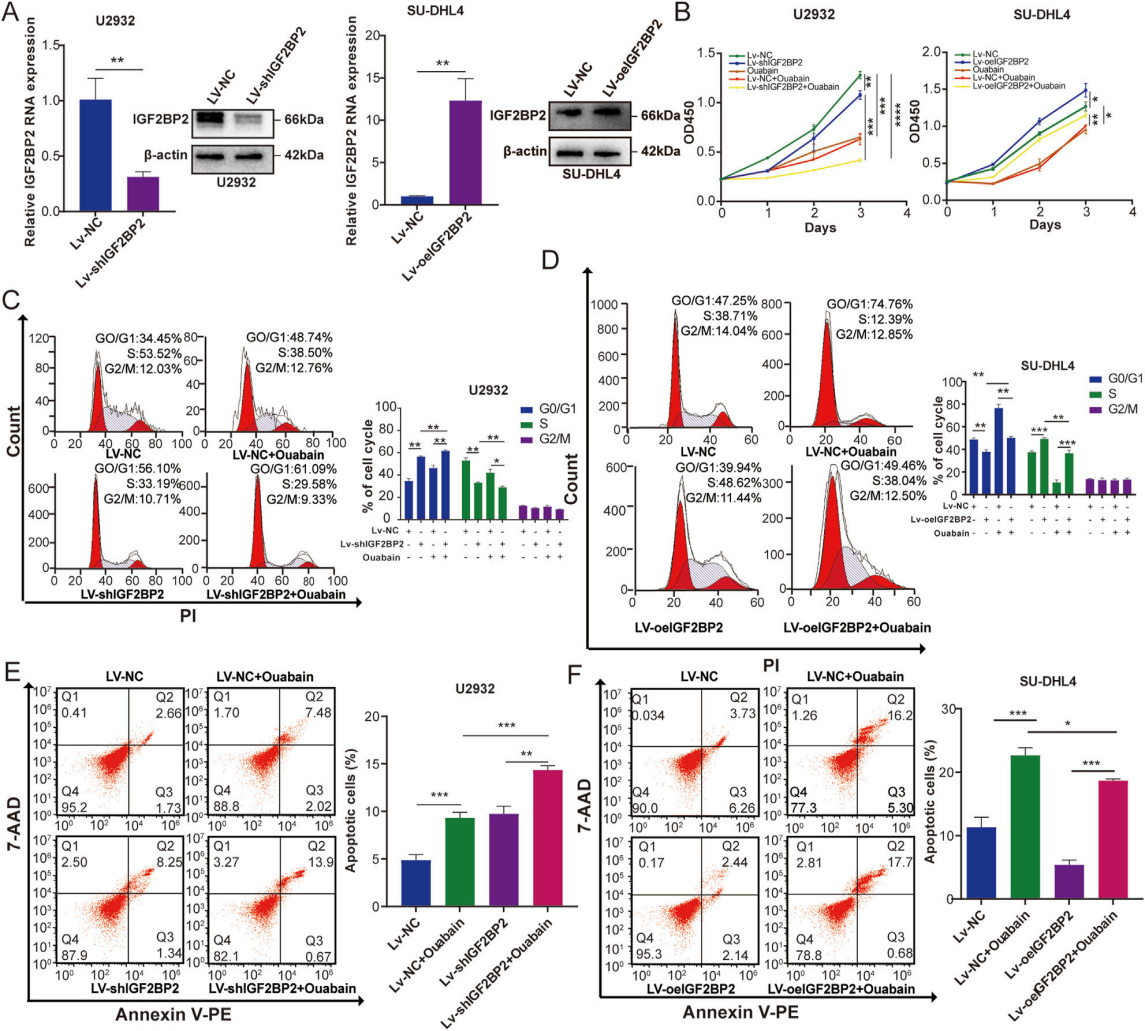
IGF2BP2 affects the sensitivity of DLBCL cell lines to ouabain. **(A)** The effect of IGF2BP2 knockdown in U2932 cells and the effect of IGF2BP2 overexpression in SU-DHL4 cells were assessed through quantitative PCR (qPCR) and western blotting (WB). **(B)** A Cell Counting Kit-8 assay was utilized to assess the proliferation of U2932 and SU-DHL4 cells treated with ouabain. **(C)** IGF2BP2 knockdown combined with ouabain treatment significantly induced cell cycle arrest at the G1/S phase in U2932 cells, as assessed by flow cytometry. **(D)** IGF2BP2 overexpression inhibited ouabain-induced cell cycle arrest at the G1/S phase in SU-DHL4 cells, as assessed by flow cytometry. **(E)** IGF2BP2 knockdown combined with ouabain treatment significantly promoted apoptosis in U2932 cells, as assessed by flow cytometry. **(F)** IGF2BP2 overexpressed inhibits ouabain-induced apoptosis in SU-DHL4 cells, as assessed by flow cytometry. The data are presented as the means ± SDs; n = 3. *p < 0.05, **p < 0.01, ***p < 0.001, ****p < 0.0001 (Student’s t-test).

IGF2BP2 plays an important role as a m^6^A-modified reading protein in regulating cellular mRNA localization, stability, and translation (Wang et al., 2021). To investigate the effect of IGF2BP2 on DLBCL cells under the influence of ouabain, we overexpressed IGF2BP2 in SU-DHL4 cells and downregulated IGF2BP2 in U2932 cells (Figure 6A). We then treated these two cell lines with ouabain Cell proliferation in the Lv-shIGF2BP2+Ouabain group was significantly inhibited according to the results of the CCK-8 assay (Figure 6B). Flow cytometry analysis revealed that the cell cycle of the Lv-shIGF2BP2+Ouabain group was inhibited considerably in the G1/S phase (Figure 6C). Furthermore, IGF2BP2 knockdown considerably increased the degree of apoptosis induced by ouabain treatment (Figure 6E). Conversely, when ouabain was administered to the IGF2BP2-overexpressing cell lines, the CCK-8 method resulted in reduced inhibition of cell proliferation and cell cycle arrest in the Lv-oeIGF2BP2+Ouabain group, along with decreased levels of apoptosis compared with those observed in the Lv-NC + Ouabain group (Figure 6B right, Figures 6D, F). These results suggest that knockdown of IGF2BP2 enhances the sensitivity of DLBCL cells to ouabain.

## 4 Discussion

DLBCL is a monoclonal proliferative malignant tumor with heterogeneity and aggressiveness, and its incidence is increasing year annually (Chapuy et al., 2018). Epigenetic modifications are important drivers of the development of hematologic malignancies. Among them, N6-methyladenosine (m^6^A) methylation is a key epigenetic modification that controls a variety of fundamental biological processes (Wang et al., 2015). For example, high expression of IGF2BP3 is associated with a proliferative phenotype of pediatric B-cell acute lymphoblastic leukemia (B-ALL) at the mRNA and protein levels and portends favorable survival in high-risk B-ALL patients (Mäkinen et al., 2021). ALKBH5 is a demethylase and increasing evidence has shown that ALKBH5 is closely related to tumor growth, proliferation, and survival. Studies have shown that ALKBH5 regulates the PI3K/AKT pathway by regulating the stability of AXL mRNA (Wang et al., 2020).In addition, high expression of ALKBH5 is associated with poor prognosis in AML patients. IGF2BP2 is a carcinomatous fetal gene that is expressed at lower levels in normal adult tissues than in fetal liver tissues and is overexpressed in various types of cancer, such as glioblastoma, liver cancer, and breast cancer, thus making it a promising therapeutic target in cancer (Hammer et al., 2005; Weng et al., 2022). In particular, Weng et al. (2022) reported the oncogenic role and therapeutic targeting of the reading protein IGF2BP2 in AML (Weng et al., 2022). Research on m^6^A modification is important in the study of DLBCL pathogenesis. Our study revealed that ALKBH5 and IGF2BP2 may serve as novel therapeutic targets for the treatment of DLBCL.

Cardiac glycosides can not only inhibit the growth of some cancer cells but also reduce their recurrence and metastasis (Mijatovic et al., 2007; Laverdière et al., 2018; Zheng et al., 2021). Ouabain is a cardiotonic steroid drug derived from digitalis, that is mainly used to treat heart failure (Xiao et al., 2017). In recent years, researchers have reported that ouabain can promote the apoptosis of lung cancer (Chanvorachote and Pongrakhananon, 2013), acute myeloid leukemia (Valdes et al., 2007; Tailler et al., 2011), liver cancer (Ozdemir et al., 2012), and other tumor cells. These findings suggest that ouabain may be effective against a variety of hematological tumors, including DLBCL. In our previous study, the addition of cardiac glycosides (digoxin) to 3 patients with DLBCL was found to prolong the survival of patients. This led us to explore the potential mechanism of action of cardiac glycosides, such as ouabain, in patients with DLBCL. To clarify the effect of ouabain on DLBCL progression, we treated OCI-LY3 cells with ouabain and performed RNA sequencing, which revealed that differentially expressed genes associated with cell proliferation, and apoptosis were significantly enriched after ouabain treatment. Further verification experiments revealed that ouabain significantly inhibited the proliferation and accelerated the apoptosis of DLBCL cells. These results suggest that ouabain may be a candidate for the treatment of DLBCL. Therefore, elucidating the target of action where ouabain may act in the treatment of DLBCL has become a critical issue that needs to be urgently addressed.

After RNA sequencing of the DLBCL cell line OCI-LY3 and detection of m^6^A-modifying enzymes, we observed that, compared with the control group, the ouabain treatment group was significantly involved in the progression of RNA degradation, which was affected by m^6^A methylation. m^6^A methylation is associated with the development of multiple tumors, and ouabain has been shown to regulate tumor suppressor genes through epigenetic mechanisms (Raynal et al., 2016; Yang et al., 2020; Song et al., 2022). Epigenetic m^6^A isomerization is regulated by regulators such as methyltransferases, demethylases, and reading enzymes (Zhang et al., 2020; Oerum et al., 2021). For example, the m^6^A-binding protein IGF2BP2 is highly expressed in acute myeloid leukemia (AML) and is associated with poor prognosis (Weng et al., 2022). N^6^-methyladenosine modification of TRERNA1 mediated by the m^6^A demethylase ALKBH5 promotes DLBCL proliferation by downregulating p21 (Song et al., 2022). In addition, Chen et al. found that YTHDF2 is involved in the progression and poor prognosis of DLBCL. By enhancing the stability and expression of ACER2, YTHDF2 triggers endogenous ceramide catabolism, increases S1P levels, and activates the PI3K/AKT and ERK pathways. This study highlights the potential of YTHDF2 as a therapeutic target and predictor of DLBCL (Chen et al., 2023b).Some studies have shown that m^6^A shows different heterogeneity in most tumors. Cancer type-specific m^6^A levels regulate the expression of different cancer-related genes in different cancer types (Lin et al., 2024). We treated DLBCL cells with different concentrations of ouabain and detected the expression levels of m^6^A-related modification enzymes. Our findings revealed a positive correlation between the expression levels of ALKBH5 and IGF2BP2 and increasing concentrations of ouabain.This interesting phenomenon leads us to think about how exactly ouabain regulates the expression of ALKBH5 and IGF2BP2. Subsequent analysis of RNA-seq data revealed the downregulation of two regulatory factors, SF3B4 and YWHAG, following ouabain treatment. SF3B4 and YWHAG have been identified as regulators of m^6^A (An et al., 2020). Therefore, we treated OCI-LY3 cells with IC50 concentration of ouabain and verified that the expression of SF3B4 and YWHAG was indeed downregulated after ouabain treatment Knockdown of SF3B4 resulted in decreased expression levels of ALKBH5 and IGF2BP2.However, no such effect was observed for YWHAG knockdown. Further validation at the protein level confirmed that SF3B4 indeed influences the expression of ALKBH5 and IGF2BP2 (Supplementary Figure S4). Notably, recent research suggests that H3K36me3 histone modification may guide the installation of classically enriched m^6^A modifications near stop codons by directly recruiting METTL14 (Huang et al., 2019). Therefore, it is hypothesized that ouabain affects the expression of ALKBH5 and IGF2BP2 through modulation of SF3B4-mediated m^6^A regulation. Next, we aimed to explore whether ALKBH5 and IGF2BP2 are involved in the development of DLBCL. Through analysis via the GEO database and examination of tissues from DLBCL patients, it was evident that both ALKBH5 and IGF2BP2 were highly expressed in DLBCL patients compared with normal controls. Subsequently, stable cell lines which ALKBH5 and IGF2BP2 were overexpressed or knocked downwere constructed via lentivirus technology which demonstrated that overexpression significantly promoted cell proliferation, whereas knockdown resulted in a significant decrease in cell viability. In conclusion, these results suggest important roles for ALKBH5 and IGF2BP2 in DLBC development and highlight their potential as therapeutic targets for further investigation. The same dose of ouabain had a more pronounced inhibitory effect on the proliferation and cell cycle of cells in which ALKBH5 or IGF2BP2 was knocked down. In addition, ALKBH5 and IGF2BP2 upregulation significantly reversed ouabain-induced promotion of apoptosis. Conversely, when ALKBH5 and IGF2BP2 were knocked down, the apoptosis-inducing effect of ouabain on DLBCL cells was significantly enhanced. These results indicate that ouabain affects the malignant progression of DLBCL by influencing the m^6^A demethylase ALKBH5 and the m^6^A-binding protein IGF2BP2, suggesting that ALKBH5 and IGF2BP2 may be potential targets of ouabain action.

Taken together, these results suggest that ouabain mediates epigenetic regulation for the treatment of DLBCL through the effects of the m^6^A demethylase ALKBH5 and the m^6^A-binding protein IGF2BP2, which can be used as novel biomarkers for the diagnosis of DLBCL. This study provides new theoretical support and targeted therapeutic strategies for the treatment of DLBCL.

## Data Availability

The original datasets used in this study are available in the Gene Expression Omnibus (GEO) database (https://www.ncbi.nlm.nih.gov/geo/query/acc.cgi?acc=GSE12453, https://www.ncbi.nlm.nih.gov/geo/query/acc.cgi?acc=GSE83632). Further inquiries can be directed to the corresponding authors.
