# Supplementary material for: Ouabain-mediated downregulation of ALKBH5 and IGF2BP2 inhibits the malignant progression of DLBCL
Source: Front Pharmacol. 2024 Aug 30;15:1447830. doi: 10.3389/fphar.2024.1447830 (PMC11392878; doi:10.3389/fphar.2024.1447830)

Supplementary Material

Ouabain-Mediated Downregulation of ALKBH5 and IGF2BP2 Inhibits the Malignant Progression of DLBCL

Yuxin Hong[1†], Hehua Ma[2†], Haoyi Yang[1], Yuning Zhu[1], Yuan Wei[1], ZhenzhenXu[2], Yuwen Zhang[3], Dandan Jin[1], Zhiyou Chen[1], Wei Song[2]*, Juan Li[1]*

[1]Department of Phase I Clinical Trials Unit, Nanjing Drum Tower Hospital Clinical College of Nanjing University of Chinese Medicine, Nanjing, 210023, China.

[2]Phase I Clinical Trials Unit, Nanjing Drum Tower Hospital, Affiliated Hospital of Medical School, Nanjing University, Nanjing, 210008, China.

[3]Department of Phase I Clinical Trials Unit, China Pharmaceutical University Nanjing Drum Tower Hospital, Nanjing, 211198, China.

[†]These authors contributed equally to this work and shared the first authorship

***Correspondence:**

Corresponding Author

Juan Li*, juanli2003@163.com.

Wei Song*, songwei3929@163.com.

1 Supplementary Figures and Tables

1.1.Supplementary Figures

Supplementary Figure 1: Original gels for Western blots in Figure 5A.

The original Western blot images of β-actin. From left to right: Marker, Lv-NC, Lv-oeALKBH5/ Lv-shALKBH5.The molecular weight of β-actin is 42 kDa.

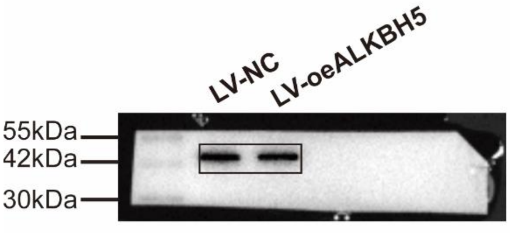

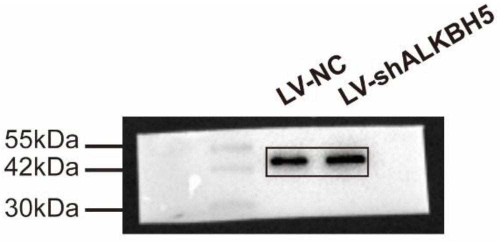

The original Western blot images of ALKBH5. From left to right: Marker, Lv-NC, Lv-oeALKBH5/ Lv-shALKBH5.The molecular weight of ALKBH5 is 44 kDa.

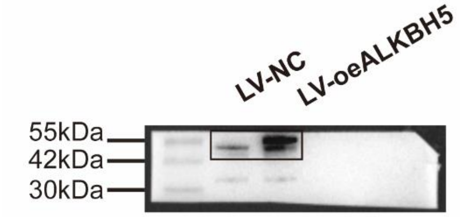

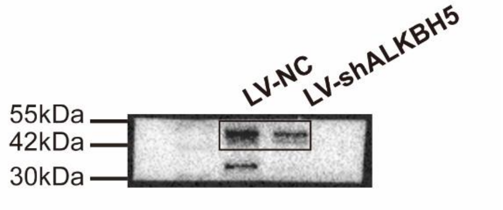

Supplementary Figure 2: Original gels for Western blots in Figure 6A.

The original Western blot images of β-actin. From left to right: Marker, Lv-NC, Lv-oeIGF2BP2/ Lv-shIGF2BP2.The molecular weight of β-actin is 42 kDa.

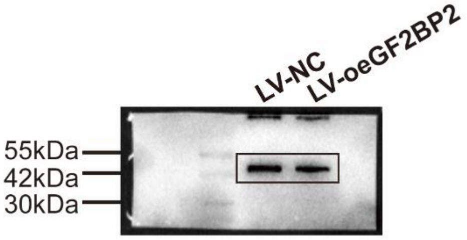

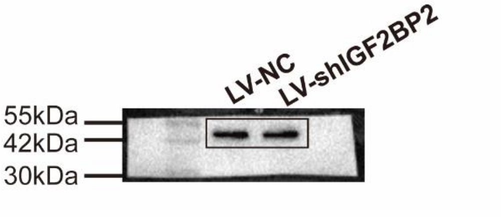

The original Western blot images of IGF2BP2. From left to right: Marker, Lv-NC, Lv-oeIGF2BP2/ Lv-shIGF2BP2.The molecular weight of ALKBH5 is 66 kDa.

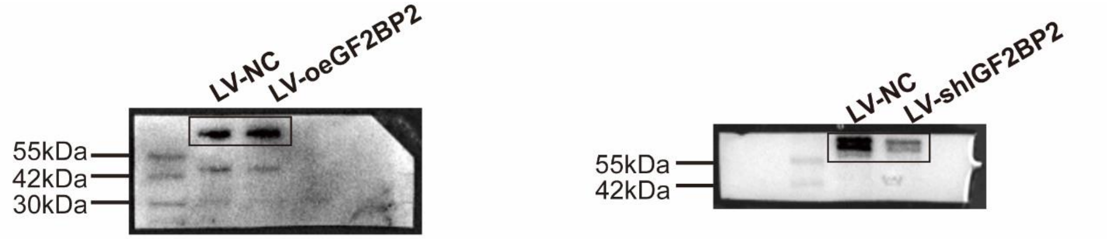

Supplementary Figure 3: Original gels for Western blots in Figure S2A.

The original Western blot images of β-actin, ALKBH5 and IGF2BP2. The molecular weight of β-actin, ALKBH5, and IGF2BP2 is 42 kDa, 44 kDa and 66 kDa.

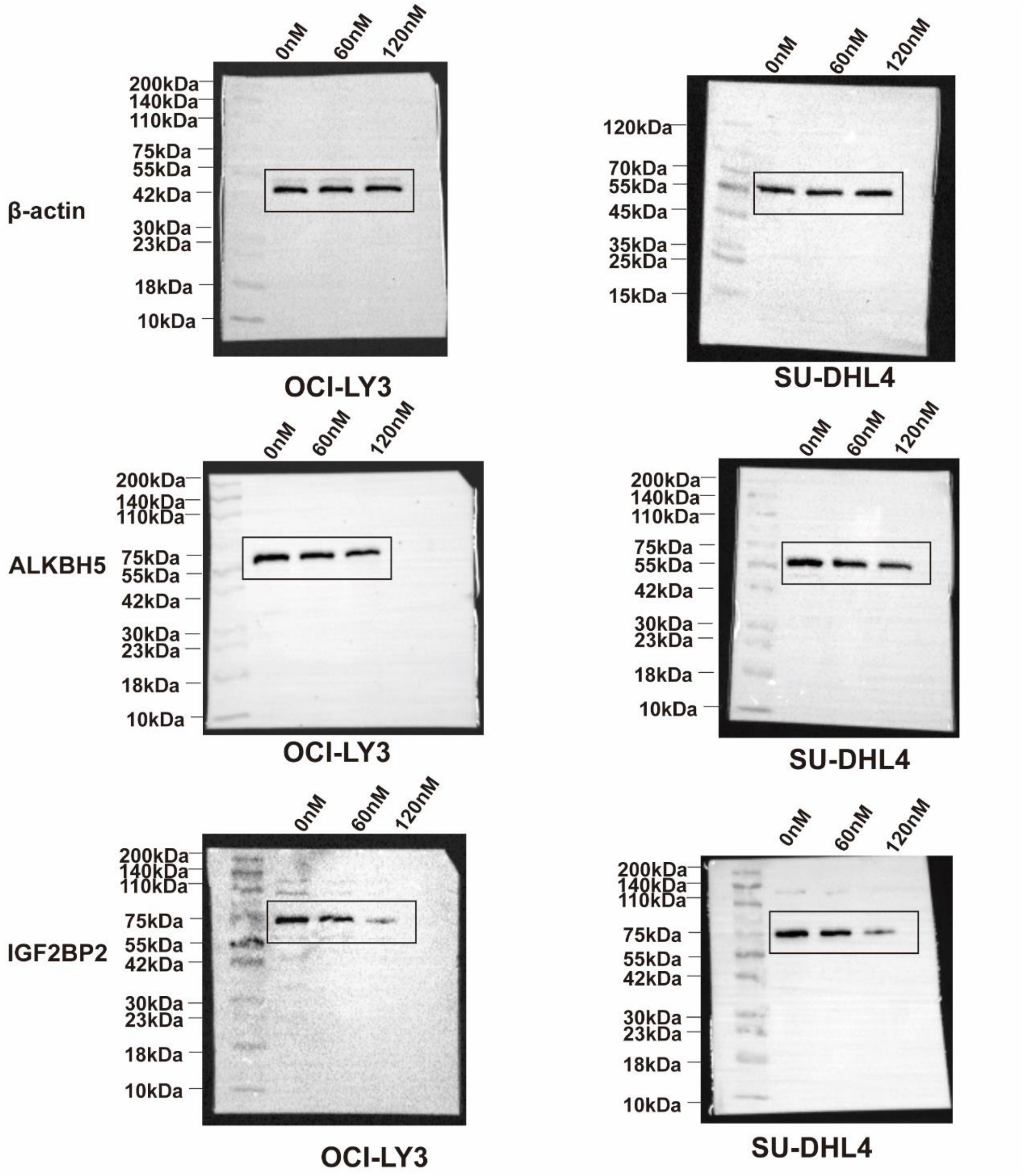

Supplementary Figure 4: Original gels for Western blots in Figure S4A.

The original Western blot images of β-actin, SF3B4 and YWHAG. The molecular weight of β-actin, ALKBH5, and IGF2BP2 is 42 kDa, 49 kDa and 28 kDa.

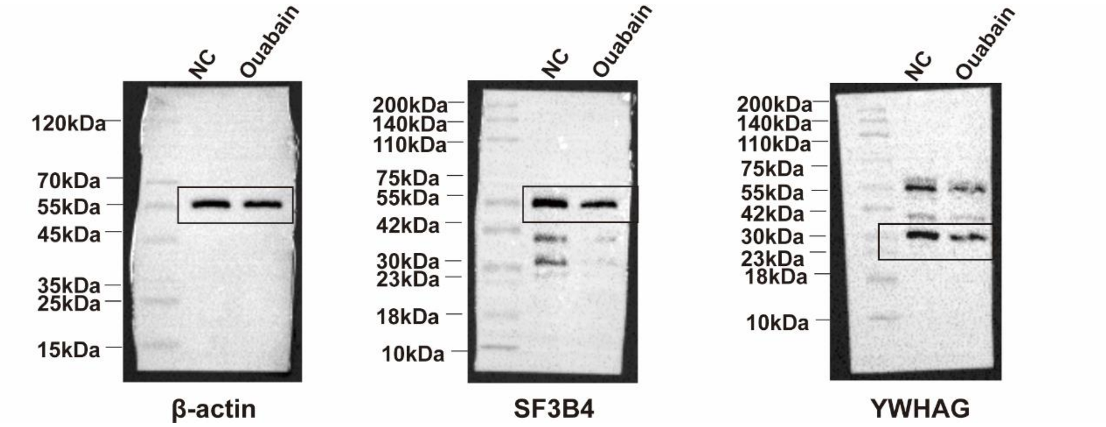

Supplementary Figure 5: Original gels for Western blots in Figure S4C.

The original Western blot images of β-actin, ALKBH5 and IGF2BP2. The molecular weight of β-actin, ALKBH5, and IGF2BP2 is 42 kDa, 44kDa and 66 kDa.

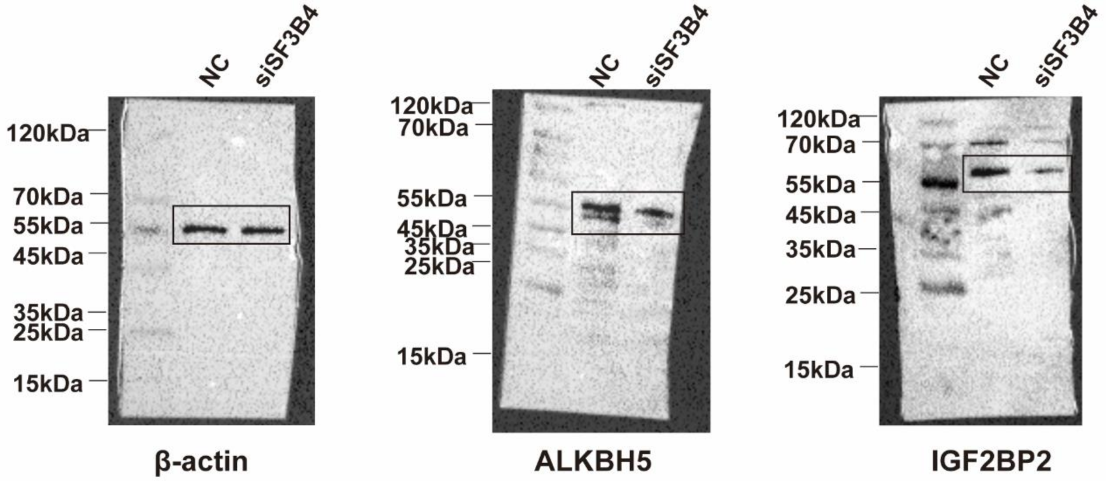

Supplement: Supplementary file 1 [file DataSheet2.pdf]
