# Supplementary material for: Ouabain-mediated downregulation of ALKBH5 and IGF2BP2 inhibits the malignant progression of DLBCL
Source: Front Pharmacol. 2024 Aug 30;15:1447830. doi: 10.3389/fphar.2024.1447830 (PMC11392878; doi:10.3389/fphar.2024.1447830)
Supplement: Supplementary file 2 [file DataSheet1.pdf]

Supplementary Material

Ouabain-Mediated Downregulation of ALKBH5 and IGF2BP2 Inhibits the Malignant Progression of DLBCL

Yuxin Hong[1†], Hehua Ma[2†], Haoyi Yang[1], Yuning Zhu[1], Yuan Wei[1], ZhenzhenXu[2], Yuwen Zhang[3], Dandan Jin[1], Zhiyou Chen[1], Wei Song[2]*, Juan Li[1]*

[1]Department of Phase I Clinical Trials Unit, Nanjing Drum Tower Hospital Clinical College of Nanjing University of Chinese Medicine, Nanjing, 210023, China.

[2]Phase I Clinical Trials Unit, Nanjing Drum Tower Hospital, Affiliated Hospital of Medical School, Nanjing University, Nanjing, 210008, China.

[3]Department of Phase I Clinical Trials Unit, China Pharmaceutical University Nanjing Drum Tower Hospital, Nanjing, 211198, China.

[†]These authors contributed equally to this work and shared the first authorship

***Correspondence:**

Corresponding Author

Juan Li*, juanli2003@163.com.

Wei Song*, songwei3929@163.com.

1. Supplementary Figures and Tables

1.1. Supplementary Figures

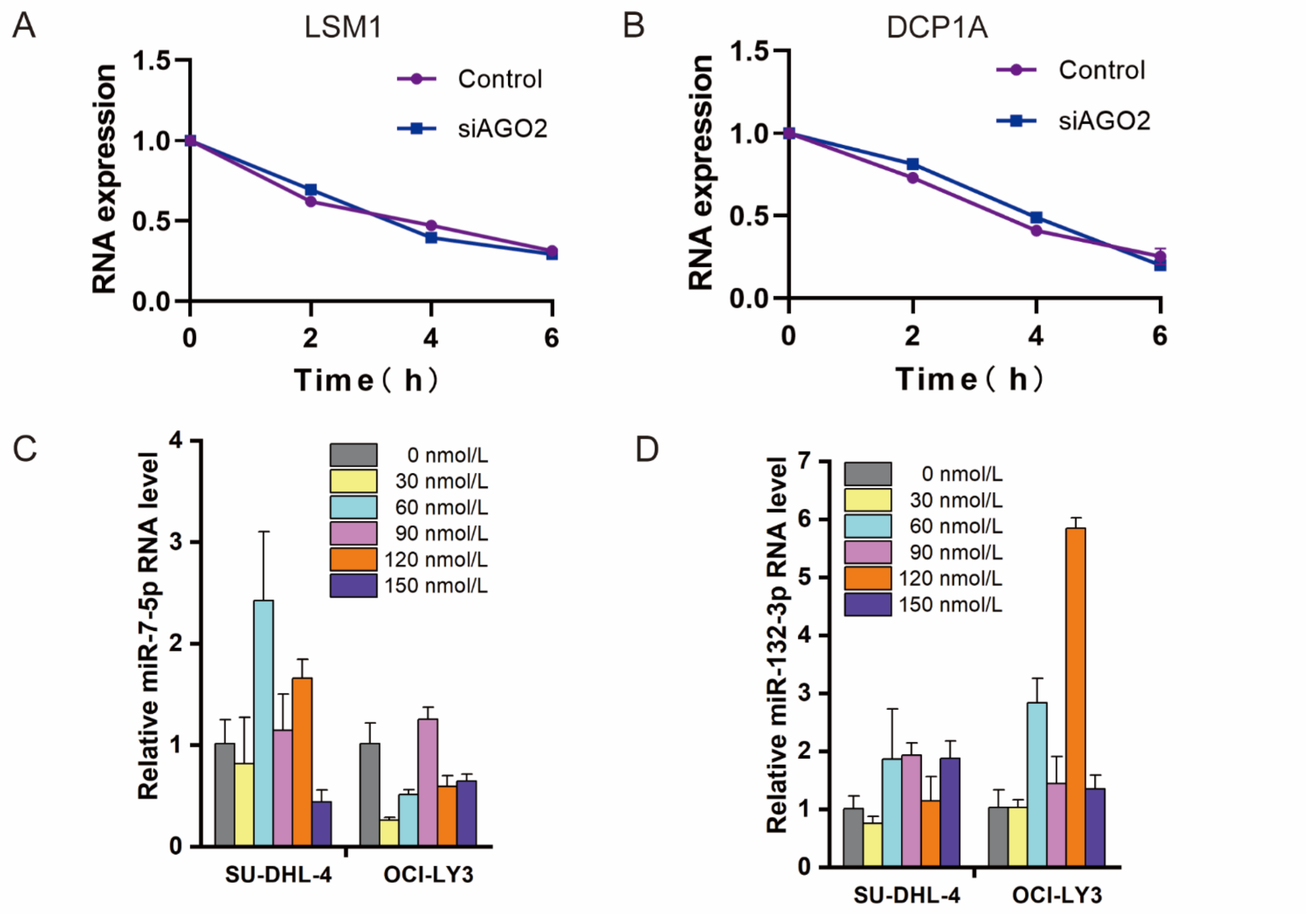

Figure S1. Effect of miRNA on RNA degradation. (A) LSM1 levels in siAGO2 and control cells were quantified by qPCR at the indicated time points after actinomycin D treatment. The cells were treated with 5 μg/ml of actinomycin D at 0, 2, 4, and 6 h. (B) DCP1A levels in siAGO2 and control cells were quantified by qPCR at the indicated time points after actinomycin D treatment. The cells were treated with 5 μg/ml of actinomycin D at 0, 2, 4, and 6 h. (C) Effects of different concentrations of ouabain on the expression of miR-7-5p were detected via q-PCR. (D) Effects of different concentrations of ouabain on the expression of miR-132-3p were detected via q-PCR.

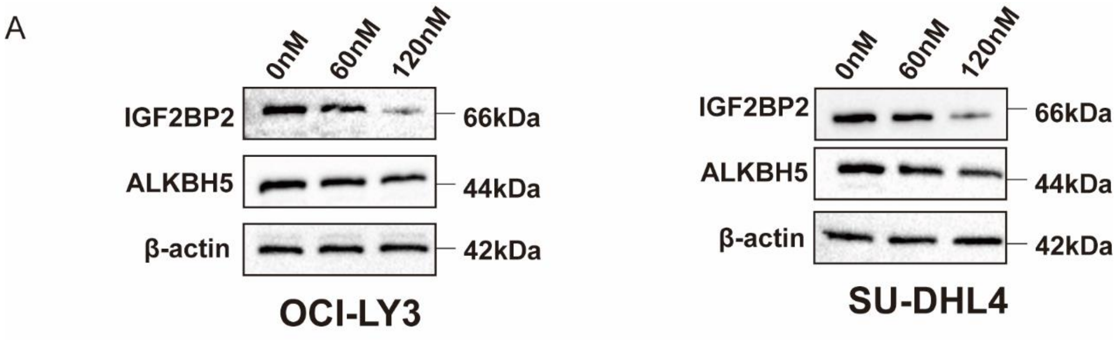

Figure S2A. Verify the effects of different concentrations of ouabain on the protein abundance of ALKBH5 and IGF2BP2 via western blot (WB).

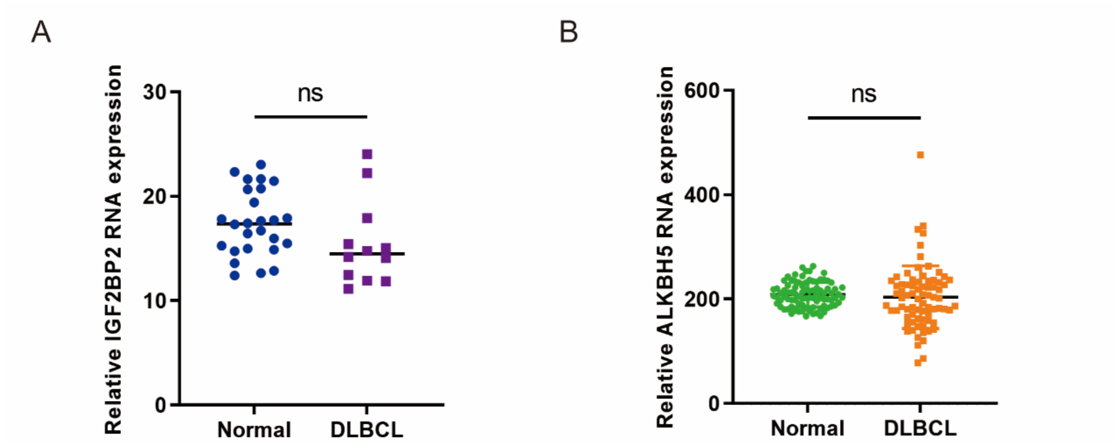

Figure S3. Gene expression analysis of ALKBH5 and IGF2BP2 in DLBCL tissues. (A) The expression level of IGF2BP2 in diffuse large B-cell lymphoma (DLBCL) was analyzed in the GSE12453 cohort. (B) The expression level of ALKBH5 in diffuse large B-cell lymphoma (DLBCL) samples from the GSE83632 cohort was analyzed. The data are presented as the means ± SDs; n = 3. ^{ns} $P > 0.05$ (Student's t-test).

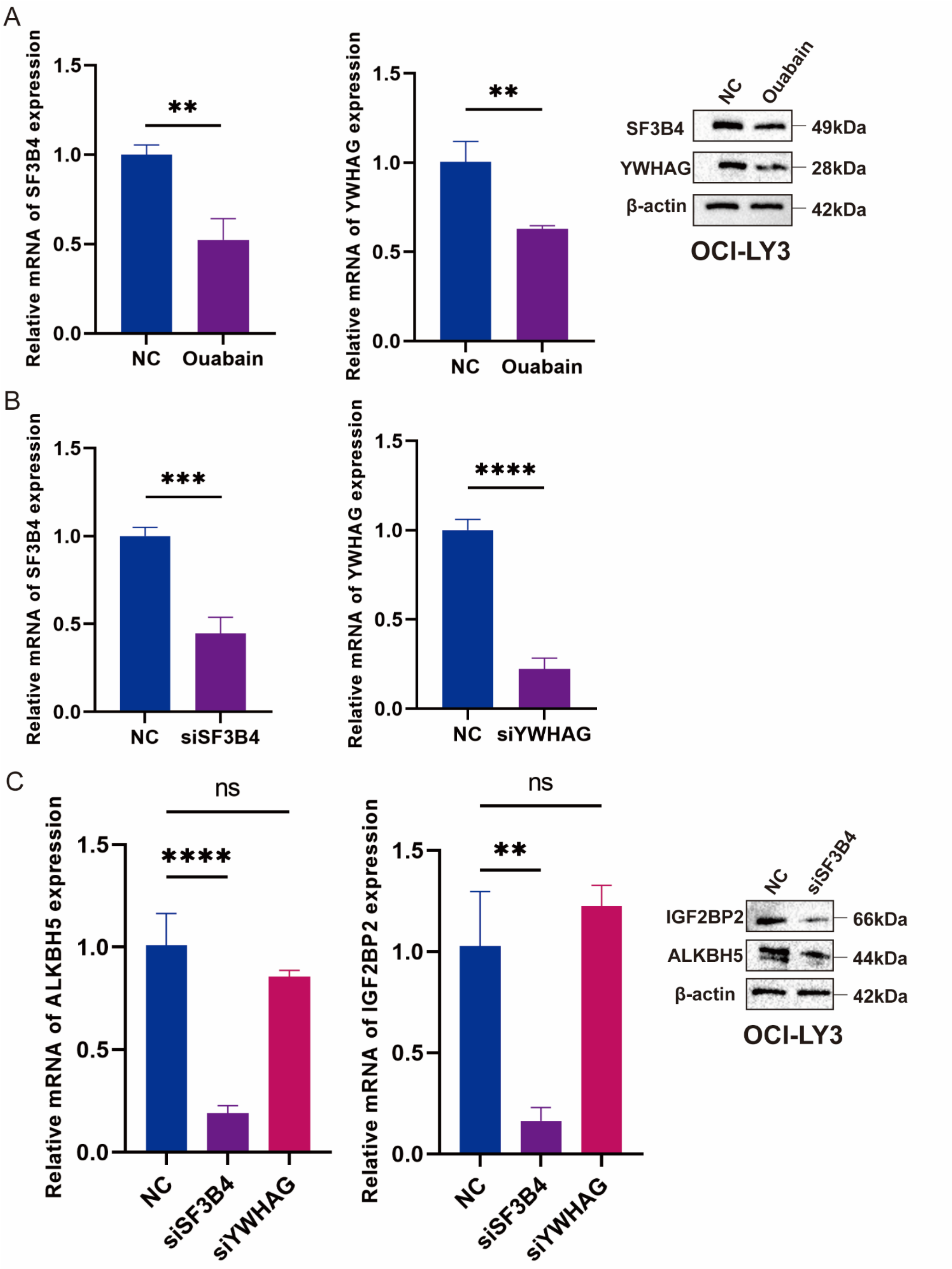
A
Relative mRNA of SF3B4 expression
1.5
1.0
0.5
0.0
**
NC
Ouabain
Relative mRNA of YWHAG expression
1.5
1.0
0.5
0.0
**
NC
Ouabain
NC
Ouabain
SF3B4
YWHAG
β-actin
49kDa
28kDa
42kDa
OCI-LY3
B
Relative mRNA of SF3B4 expression
***
NC
siSF3B4
Relative mRNA of YWHAG expression
****
NC
siYWHAG
C
Relative mRNA of ALKBH5 expression
ns
****
NC
siSF3B4
siYWHAG
Relative mRNA of IGF2BP2 expression
ns
**
NC
siSF3B4
siYWHAG
NC
siSF3B4
IGF2BP2
ALKBH5
β-actin
66kDa
44kDa
42kDa
OCI-LY3

FigureS4. Ouabain influences ALKBH5 and IGF2BP2 expression by mediating SF3B4. (A) The expression of SF3B4 and YWHAG in DLBCL cells was down-regulated by ouabain. (B) SF3B4 and YWHAG were knocked down on OCI-LY3 cells, respectively. (C) The effect of YWHAG and SF3B4 knockdown on the expression of ALKBH5 and IGF2BP2 was verified by RT-qPCR and Western blot (WB). The data are presented as the means ± SDs; n = 3. *$P < 0.05$, **$P < 0.01$, ***$P < 0.001$, ****$P < 0.0001$ (Student’s t-test).

1.2. Supplementary Tables

Table S1. siRNA sequence of genes used in this manuscript

**Gene names**	**Sequences**
siSF3B4 Sense	5′-GGAUGAGAAGGUUAGUGAATT-3′
siSF3B4 Antisense	5′-UUCACUAACCUUCUCAUCCTT-3′
siYWHAG Sense	5′-CGAUUAGGCCUGGCUCUUATT-3′
siYWHAG Antisense	5′-UAAGAGCCAGGCCUAAUCGTT-3′
shALKBH5	5′-UCAACAGCGCCGUCAUCAATT-3′
shIGF2BP2	5′-TTCTCCGAACGTGTCACGT-3′

Table S2. List of primers used in this study. Primers for quantitative real-time PCR for gene expression analysis

**Primer names**	**Sequences**
ACTIN forward	5’-GTCATTCCAAATATGAGATGCGT-3’
ACTIN reverse	5’-GCTATCACCTCCCCTGTGTG-3’
METTL3 forward	5’-AAGCTGCACTTCAGACGAAT-3’
METTL3 reverse	5’-GGAATCACCTCCGACACTC-3’
METTL14 forward	5’-AGAAACTTGCAGGGCTTCCT-3’

METTL14 reverse	5’-TCTTCTTCATATGGCAAATTTTCTT-3’
WTAP forward	5’-GGCGAAGTGTCGAATGCT-3’
WTAP reverse	5’-CCAACTGCTGGCGTGTCT-3’
IGF2BP1 forward	5’-ATCGGCAACCTCAACGAGAG-3’
IGF2BP1 reverse	5’-GTTTCGATGGCCTTCATCGC-3’
IGF2BP2 forward	5’-CTACGCCTTCGTGGACTACC-3’
IGF2BP2 reverse	5’-TGTTGACTTGTTCCACATTCTCC-3’
IGF2BP3 forward	5’-ACTGCACGGGAAACCCATAG-3’
IGF2BP3 reverse	5’-TCCCACTGTAAATGAGGCGG-3’
ALKBH5 forward	5’-CCCGAGGGCTTCGTCAACA-3’
ALKBH5 reverse	5’-CGACACCCGAATAGGCTTGA-3’
FTO forward	5’-TGGGTTCATCCTACAACGG-3’
FTO reverse	5’-CCTCTTCAGGGCCTTCAC-3’
U6 forward	5’-CGCAAGGATGACACG-3’
U6 reverse	5’-GAGCAGGCTGGAGAA-3’
miR-132-3p forward	5’- GCGCGTAACAGTCTACAGCCA -3’
miR-132-3p reverse	5’- AGTGCAGGGTCCGAGGTATT -3’
miR-7-5p forward	5’- CGCGTGGAAGACTAGTGATTTT -3’
miR-7-5p reverse	5’- AGTGCAGGGTCCGAGGTATT -3’

The relative expression of target RNA was evaluated via the comparative $2^{-\Delta\Delta Cq}$ method.
